# Structure–Function Decoupling Across Cerebrovascular Diseases: A Convergent Network Phenotype Requiring Disease‐Specific Interpretation

**DOI:** 10.1002/cns.71095

**Published:** 2026-08-14

**Authors:** Tingting Zhang, Xiaoying Zhao, Hui Zhang, Bingwei Zhang

**Affiliations:** ^1^ Department of Neurology First Affiliated Hospital of Dalian Medical University Dalian China

Dear Editor,

1

We read with great interest the recent article by Dong et al. [1] entitled “Network disconnection syndrome in unruptured brain arteriovenous malformations: a multimodal connectome study” published in ***CNS Neuroscience & Therapeutics***. Challenging traditional lesion‐centric models, the authors demonstrated that cognitive dysfunction in unruptured brain arteriovenous malformations (bAVMs) is better explained by network disconnection driven primarily by impaired long‐range white matter integrity than by focal damage. They further showed that structural disruption was accompanied by aberrant structure–function (SC–FC) decoupling in association networks (including the frontoparietal and ventral attention systems), which was associated with executive and memory deficits. Crucially, these cognitive deficits were evident despite preserved global functional topology and interhemispheric compensatory reorganization, suggesting that global compensation may be insufficient to prevent the breakdown of higher‐order circuits. Given that unruptured bAVMs are rare, these findings highlight an important yet underrecognized clinical need: to extend investigation beyond motor symptoms toward cognitive, emotional, and behavioral domains—dimensions that profoundly affect quality of life yet remain largely unaddressed in rare cerebrovascular diseases. With the rapid advancement of high‐resolution multimodal imaging, systematically capturing these “silent” non‐motor consequences should become a research priority.

These findings raise the possibility that SC–FC decoupling may represent a convergent systems‐level phenotype across heterogeneous cerebrovascular conditions. In cerebral small vessel disease (CSVD), voxel‐wise and network‐level decoupling correlates with disease burden and mediates cognitive dysfunction, particularly within the cognitive control network [2, 3]. In cognitively normal individuals with white matter hyperintensities (WMHs), altered SC–FC coupling may represent a candidate preclinical imaging marker [4]. In acute and chronic ischemic stroke, node‐specific and pathway‐selective SC‐FC alterations have been associated with motor and cognitive impairment and predict functional recovery [5, 6, 7]. However, similar SC–FC abnormalities do not necessarily imply a single shared biological mechanism. In bAVMs, chronic high‐flow shunting, venous hypertension, hemodynamic steal, and perinidal congestion may alter white matter integrity and functional signals [1]; CSVD is characterized by diffuse arteriolar pathology, chronic hypoperfusion, and white matter degeneration [2, 3]; ischemic stroke involves acute tissue and tract destruction followed by diaschisis and time‐dependent functional reorganization [7, 8]. Thus, the cross‐disease similarity may reflect phenotypic convergence at the systems level rather than mechanistic equivalence. The shared denominator may be reduced correspondence between anatomical architecture and functional network dynamics, whereas the upstream drivers and the biological meaning and clinical interpretation of a given coupling value remain disease‐ and stage‐specific. Accordingly, SC–FC decoupling may be best regarded as a candidate transdiagnostic network readout rather than a mechanism‐independent universal biomarker.

Structural disconnection and SC–FC decoupling should also be distinguished conceptually and methodologically. Structural disconnection refers to the reduction or interruption of anatomical pathways, typically quantified using tract integrity, structural edge weights, or derived network‐topological measures [8]. By contrast, SC–FC decoupling is a relational measure describing reduced correspondence between structural and functional connectivity patterns [9]. The former can contribute to the latter, but they are not interchangeable. After acute ischemic stroke, structural disconnection may precede detectable functional reorganization, so statistically significant decoupling may not yet be apparent [7, 8]. Conversely, diaschisis, compensatory synchronization, neurovascular uncoupling, or hemodynamic effects may alter functional connectivity and generate decoupling even when gross structural connectivity appears relatively preserved [7]. SC, FC, and their coupling should therefore be reported separately and interpreted according to disease stage, particularly in longitudinal and cross‐disease comparisons.

Despite these convergent findings, several critical gaps remain. Methodologically, heterogeneity in quantifying SC–FC coupling—spanning voxel‐wise, nodal, network, and pathway‐specific levels—limits cross‐disease comparison and the establishment of harmonized, disease‐calibrated reference ranges. Temporally, existing evidence, including the study by Dong et al. [1] is predominantly cross‐sectional, restricting insight into dynamic evolution. It remains unknown whether decoupling reflects irreversible structural injury, transient hemodynamic disturbance, or potentially remediable maladaptive plasticity. Mechanistically, the relative contributions of white matter injury, myelin breakdown, neurovascular uncoupling, hemodynamic stress, and functional reorganization remain incompletely clarified. Clinically, no validated imaging pipelines, quantitative thresholds, or consensus guidelines exist to incorporate decoupling metrics into risk stratification, and targeted interventions to restore SC–FC alignment remain largely unexplored.

Overcoming these barriers requires a shift from observation to mechanistic and interventional validation. Standardized, reproducible, and harmonized decoupling metrics should be validated in large multicenter longitudinal cohorts. Longitudinal designs should track SC, FC, SC–FC coupling, cognition, and perfusion at clinically meaningful intervals—pre‐treatment, early post‐treatment, 3 months, and 12 months after microsurgery or embolization, and baseline, 6, 12, and 24 months after stereotactic radiosurgery. These trajectories could distinguish re‐coupling from persistent decoupling or transient treatment‐related worsening. Causal models are needed to determine how tract‐specific injury, hemodynamic stress, and functional reorganization converge on similar topological readouts, while interventional work should evaluate whether connectome‐guided surgical planning, neuromodulation, or rehabilitation can restore structure–function alignment. Advanced diffusion MRI may further clarify the microstructural basis of decoupling. In the specific hemodynamic context of bAVMs, the Neurite Orientation Dispersion and Density Imaging (NODDI) isotropic volume fraction (isovf) may be the most immediately informative in perinidal tissue because it could help test whether venous congestion and microscopic edema contribute to increased isotropic water and reduced conventional fractional anisotropy [10]. Intracellular volume fraction (ICVF) may provide complementary sensitivity to neurite density in remote long‐range tracts, whereas ODI may help separate altered orientation dispersion from reduced neurite density in regions with complex fiber architecture. These parameters should therefore be interpreted jointly using prospective multi‐shell acquisitions and as complements to, rather than replacements for, tractography‐based connectome measures.

In conclusion, we commend Dong et al. [1] for demonstrating that cognitive impairment in unruptured bAVMs is more closely associated with network‐level white matter disconnection than with focal lesion burden. Their observation of SC–FC decoupling converges with evidence from chronic microvascular disease and ischemic stroke, suggesting a candidate systems‐level marker of cerebrovascular network injury. However, similar topological abnormalities may arise through distinct biological mechanisms and require disease‐ and stage‐specific interpretation. A connectome‐based framework can refine risk stratification and function‐preserving interventions, but standardized longitudinal assessment of structural connectivity, functional dynamics, hemodynamics, and cognition will be essential before SC–FC decoupling can be translated into a clinically useful transdiagnostic biomarker.

## Funding

This work was supported by Liaoning Science and Technology Joint Program (Grant No. 2024‐MSLH‐082) and Xingliao Talents Program‐Distinguished Medical Expert (Grant No. XLYC2412084).

## Conflicts of Interest

The authors declare no conflicts of interest.

## Linked Article

This article is linked to Dong et al. papers. To view this article, visit https://doi.org/10.1002/cns.70819.

## Data Availability

Data sharing not applicable to this article as no datasets were generated or analysed during the current study.
